# Neonatal jaundice and developmental impairment among infants in Kilifi, Kenya

**DOI:** 10.1111/cch.12750

**Published:** 2020-02-05

**Authors:** Dorcas N. Magai, Michael Mwaniki, Amina Abubakar, Shebe Mohammed, Anne L. Gordon, Raphael Kalu, Paul Mwangi, Hans M. Koot, Charles R. Newton

**Affiliations:** ^1^ Centre for Geographic Medicine Research Coast Kenya Medical Research Institute Kilifi Kenya; ^2^ Department of Clinical, Neuro‐ and Developmental Psychology, Public Health Research Institute Vrije Universiteit Amsterdam Amsterdam The Netherlands; ^3^ Department of Clinical Research, Public Health Outcomes and Evaluation Afya Research Africa Nairobi Kenya; ^4^ Department of Public Health Pwani University Kilifi Kenya; ^5^ Institute for Human Development The Aga Khan University Nairobi Kenya; ^6^ Department of Psychiatry University of Oxford Oxford UK; ^7^ Faculty of Life Sciences and Medicine King's College London London UK

**Keywords:** child development, child disability, childhood disability, delayed language, developing countries, developmental delay

## Abstract

**Background:**

Neonatal jaundice (NNJ) is common in sub‐Saharan Africa (SSA), and it is associated with sepsis. Despite the high incidence, little has been documented about developmental impairments associated with NNJ in SSA. In particular, it is not clear if sepsis is associated with greater impairment following NNJ.

**Methods:**

We followed up 169 participants aged 12 months (57 cases and 112 controls) within the Kilifi Health Demographic Surveillance System. The diagnosis of NNJ was based on clinical laboratory measurement of total serum bilirubin on admission, whereas the developmental outcomes were assessed using the Developmental Milestones Checklist and Kilifi Development Inventory.

**Results:**

There were significant differences between the cases and controls in all developmental domains. Cases scored lower in language functioning (mean [*M*] = 6.5, standard deviation [*SD*] = 4.3 vs. *M* = 8.9, *SD* = 4.6; *p* < .001); psychomotor functioning (*Mdn* = 23, interquartile range [IQR] = 17–34 vs. *Mdn* = 31.0, IQR = 22.0–44.0; Mann–Whitney *U* = 4,122, *p* = .002); and socio‐emotional functioning ([*Mdn* = 30.0, IQR = 27.0–33.0 vs. *Mdn* = 34.0, IQR = 30.0–37.0], Mann–Whitney *U* = 4,289, *p* < .001). There was no evidence of association between sepsis and psychomotor (*r*_pb_ = −.2, *p* = .214), language (*r*_pb_ = −.1, *p* = .510), and socio‐emotional functioning (*r*_pb_ = .0, *p* = .916). Significant and medium to large portions of the variance (34–64%) in the developmental outcomes among children who survived NNJ were associated with home birth, low maternal education, and feeding problems during the first days of life.

**Conclusions:**

NNJ is associated with developmental impairments in the early childhood years; however, NNJ associated with sepsis does not lead to more severe impairment. Prenatal and postnatal care services are needed to reduce the negative impact of NNJ for children from low resourced settings.

Key messages

- Children who survived neonatal jaundice (NNJ) in Kilifi County Hospital, Kenya have poor developmental outcomes, which manifest early in life.
- NNJ associated with sepsis does not appear to aggravate the poor developmental outcomes.
- Home birth, unskilled birth attendant, low maternal level of education, and feeding problems are the main risk factors associated with poor developmental outcomes in survivors of NNJ.

## 1 INTRODUCTION

Neonatal jaundice (NNJ) is one of the leading causes of newborn hospital readmissions in the world (Slusher & Olusanya, 2012). Unlike in the western world, more children in sub‐Saharan Africa die due to poor maternal and child healthcare (Peters et al., 2008). In Kilifi County Hospital (KCH) in Kenya, NNJ was the primary diagnosis in 17% of 1,080 neonatal admissions in 1999–2001, and 24% of these children died in the hospital (English et al., 2003). NNJ occurs as a result of increased bilirubin production and a reduced excretory capacity caused by immaturity of the liver (English et al., 2003; Gordon, English, Dzombo, Karisa, & Newton, 2005).

Elevated levels of bilirubin (hyperbilirubinaemia) are associated with adverse developmental outcomes including cerebral palsy (disorders affecting an individual's ability to move and maintain balance), neurocognitive impairment (limitations in mental function), language processing disorders, and deafness (Gordon et al., 2005; Maimburg, Bech, Væth, Møller‐Madsen, & Olsen, 2010; Poole et al., 2003; Slusher & Olusanya, 2012). Neonatal sepsis is associated with poor developmental outcome (Gordon et al., 2005; Stoll et al., 2004) and is a common risk factor for NNJ (Garcia & Nager, 2002; Linder et al., 1988; Shahian, Rashtian, & Kalani, 2012), but it is unclear if it aggravates the impairments associated with NNJ.

In Africa, few studies have documented the impact of NNJ on childhood developmental outcomes (Gordon et al., 2005; Ogunlesi, Dedeke, Adekanmbi, Fetuga, & Ogunfowora, 2007; Olusanya, Akande, Emokpae, & Olowe, 2009; Owa & Dawodu, 1990; Wolf, Wolf, Beunen, & Casaer, 1999; Wolf, Beunen, Casaer, & Wolf, 1997). These studies have consistently demonstrated an association between NNJ and adverse neurodevelopmental outcomes. In a community‐based population study in Nigeria involving 5,262 children 0 to 3 months old, Olusanya et al. (2009) found that of the 351 with NNJ, 9% of infants who received phototherapy and 17% of infants who received exchange transfusion developed sensorineural hearing loss.

A prospective study in Zimbabwe examining the relationship between total serum bilirubin (TSB) and neurodevelopmental outcomes in 4‐month‐old infants reported that half of the 45 surviving neonates who had TSB > 400 μmol/L developed irreversible neurological impairment (limitations in the nervous system resulting in abnormal body function) at 4 months (Wolf et al., 1997). In another follow‐up study of NNJ in Kilifi, Kenya, Gordon et al. (2005) assessed 23 subjects with hyperbilirubinaemia and 24 infants with sepsis 18–32 months after birth. The authors reported that the survivors of NNJ or sepsis had more neurological impairment, developmental impairment (life‐long problems that affect everyday life function), and motor impairment (limitations in the nervous system that cause uncontrollable body movements) than the control group.

However, these studies either had small sample sizes or did not investigate the maternal and perinatal risk factors associated with the poor outcomes among survivors of NNJ. In particular, it is unclear whether NNJ associated with sepsis leads to additional impairments. Given the limitations of previous studies examining developmental outcomes related to NNJ and the existing research gaps, the current study set out to investigate the developmental outcomes and perinatal risk factors associated with poor developmental outcomes in children who survived NNJ.

## 2 METHODS

### 2.1 Participants

This study was part of a longitudinal study that was conducted at the KCH (formerly referred to as the Kilifi District hospital), to determine the causes and neurodevelopmental outcomes and identify potential intervention strategies, for neonates less than 30 days old admitted with severe hyperbilirubinaemia. During the period we conducted this study (2006–2012), we measured TSB in all neonates with a diagnosis of NNJ admitted to KCH. The incidence of bilirubin encephalopathy on admission was 3.3%. At admission to the hospital, 147 participants were randomized to a clinical trial where they received either albumin plus phototherapy (*n* = 72) or saline plus phototherapy (*n* = 75). The median age at admission was 5 (interquartile range [IQR] = 3–6) days.

The inclusion criteria at 12 months included an admission at KCH at age 30 days or less, written consent to participate in the study, discharged home alive, and being alive at 12 months. Seventeen participants died during treatment, whereas six died after discharge. More information on the cases in this study are reported by Magai et al. (2019).

During recruitment, infants with NNJ were matched (age and sex) with community controls randomly chosen from the Kilifi Health and Demographic Surveillance System (KHDSS) in the ratio of 1:2. The KHDSS is a surveillance system that covers an area of 891 km^2^ with an approximate population of 265,000 residents and contains information about the location of the households, demographic information, and residents' immigration and outmigration information. The residents are also matched with the patients register at the hospital at various hospital entry points, and the master KHDSS database is updated weekly. The inclusion criteria for controls included written informed consent, no history of hospital admission, and not being sick during assessment. All the controls who were recruited in the study came for the assessment at 12 months.

### 2.2 Diagnosis

The diagnosis of NNJ was based on clinical laboratory measurement of TSB as well as medical history and examination. During phototherapy, the age of the neonates in hours or days and their weight were considered as per the National Institute for Health and Care Excellence guidelines (Rennie, Burman‐Roy, & Murphy, 2010). The TSB values were plotted on a range lower than the baby's age or weight. Total bilirubin was measured in every neonate admitted to the hospital. Blood was obtained from the neonates using venipuncture and collected in BD Vacutainer blood collection tubes, which were shielded from sunlight. The TSB levels were measured at KCH laboratories using a photometric analyser (ILab Aries, Italy). Severe hyperbilirubinaemia was defined by a total bilirubin level > 250 μmol/L according to the World Health Organization (WHO, 1999) guidelines. Neonatal sepsis was defined as a positive blood culture in newborns in the first month of life. Laboratory investigations included blood culture and cerebrospinal fluid. Investigations of blood culture entailed the use of a minimum of 1 ml of blood obtained from each site for inoculation in the BD Bactec Pediatrics Plus/F aerobic bottle (Bactec 9050‐USA) after skin cleansing. A positive culture was considered if a recognized pathogen was isolated from the blood culture.

### 2.3 Management of NNJ

Neonates were screened for sepsis and treated with intravenous benzylpenicillin and gentamicin according to the WHO (2013) guidelines. In neonates weighing ≤2.5 kg with TSB levels from 85 μmol·L^−1^·kg^−1^ received phototherapy, and exchange transfusion was prescribed if TSB rose above 170 μmol·L^−1^·kg^−1^. In term neonates weighing above 2.5 kg, exchange transfusion was offered if TSB levels rose above 400 μmol/L. However, in “sick” neonates, management of NNJ started if TSB levels were at 30 μmol/L below these levels. Sickness was defined as abnormal temperature or respiratory rate, cough, poor feeding, abnormally sleepy or difficult to wake, convulsions, or fever (WHO, 1999). During phototherapy, 46% of the participants were randomized to receive saline, whereas 54% received 20% albumin as part of a clinical trial, but there were no significant differences in the developmental outcomes between the participants randomized in the two arms (Magai et al., 2019).

### 2.4 Neurological and developmental assessment

Anthropometric data (weight, height, head circumference, and mid‐upper arm circumference) were obtained following WHO (2007) guidelines. Additionally, eye examination for optokinetic movements (vertical and horizontal), pursuits, saccades, and any gaze abnormalities were assessed at 12 months by a clinician blinded to the participants' developmental assessments. Neurological and motor assessments were performed using a clinical evaluation proforma designed for this study. The Kilifi Development Inventory (KDI; Abubakar, Holding, et al., 2008) was used to measure psychomotor functioning. The KDI is a locally developed measure of locomotor and eye–hand coordination as observed by a trained assessor through the interaction with the child. Scores from the two domains are then added to provide a single psychomotor score. Higher scores indicate a higher level of psychomotor functioning. The KDI test has excellent reliability (Cronbach's *α* = .8–.9) and has good sensitivity to neurodevelopmental disorders, *t*(113) = 513, *p* < .001, and variations in performance (Abubakar, Holding, et al., 2008).

The Developmental Milestones Checklist (DMC) was used to measure language and socio‐emotional functioning. The DMC is a locally developed measure of developmental outcomes in infancy (3–24 months) as reported by parents. The DMC tool has 11 items that measure language functioning and 27 items that measure a child's socio‐emotional function. The primary caregiver is then asked to rate the child's activity by stating whether the child is able to carry out the activity in the last 1 month (coded 2), the child has started to learn the activity (coded 1), or if the child has not yet started the activity (coded 0). The total scores for each domain give the child's level of functioning, with higher scores indicating higher levels of language or socio‐emotional function. The DMC was designed and validated in Kilifi and has sound psychometric properties (Cronbach's *α* = .6–.9, intraclass correlation coefficient = 0.9) and good sensitivity for age, *r*(85) = .8, *p* < .001 (Abubakar, Holding, Van De Vijver, Bomu, & Van Baar, 2010). The KDI and DMC were undertaken by trained assessors who were blind to the NNJ status of the participants.

Information on risk factors was obtained through comprehensive socio‐demographic and obstetric and perinatal history questionnaires administered to the primary caregivers. The available literature informed our design of the content of the questionnaire, to include potential risk factors in Kilifi, Kenya (Abubakar, Van De Vijver, et al., 2008; Kariuki et al., 2017; Mung'ala‐Odera et al., 2006). The primary caregivers of the neonates provided written informed consent.

### 2.5 Statistical analysis

Data were double entered using HTML web forms and analysed using STATA (version 15; StataCorp, 2017). Descriptive statistics include means (*M*) and standard deviation (*SD*) distribution and medians and IQRs; χ^2^ was used to compare proportions, independent Student's *t* test (NNJ vs. control group) and analysis of variance (sepsis + NNJ vs. NNJ only and the control group) for normally distributed data, and Mann–Whitney *U* (NNJ vs. control group) and Kruskal–Wallis tests (sepsis + NNJ vs. NNJ only and the control group) for non‐normal distributed data. Point biserial correlation was used to examine the associations between having sepsis or not and the developmental scores (language, psychomotor, and social–emotional functioning). Additionally, multiple linear regressions were used to investigate the associations between potential risk factors and developmental outcomes (language, psychomotor, and social–emotional functioning) as dependent variables. Maternal occupation, sex, and height‐for‐age were entered as additional control variables.

## 3 RESULTS

### 3.1 Clinical characteristics of participants

Out of the 124 participants who were given an appointment at 12 months, 57 cases were assessed at the 12‐month follow‐up. There were no significant differences in severity of NNJ (Mann–Whitney *U =* 1,792, *p* = .158), age (Mann–Whitney *U =* 1,847, *p* = .677), and gender, *χ* ^2^(1) = 0.24, *p =* .622, between the cases who were assessed versus those who did not turn up for assessment.

In this study, 169 participants (57 cases and 112 controls) were assessed. The median maternal age was 27 (IQR = 22, 32) years. The independent *t*‐test results showed significant differences in the height‐for‐age *z* scores between cases ([*M* = −2.4, *SD* = 1.8] and controls [*M* = −1.8, *SD* = 1.5], *p* = .033) and significant differences in weight‐for‐age *z* scores between the cases ([*M* = −1.1, *SD* = 0.2] and controls [*M* = −0.5, *SD* = 1.3], *p* = .008). None of the participants had a seizure disorder, and only one participant was diagnosed with moderate cerebral palsy, whereas four had nystagmus when looking at the optokinetic nystagmus sheet in a vertical presentation.

### 3.2 Risk factors and management of NNJ

The main risk factors for NNJ that were captured in this study were ABO incompatibility (42.1%) and sepsis (22.8%). All the cases (44 with NNJ and 13 with NNJ and neonatal sepsis) in this study received phototherapy. One case received phototherapy and exchange transfusion.

### 3.3 Maternal report

The results indicate that mothers of the cases had more problems such as premature labour, oedema (pre‐eclampsia), and bleeding during the pregnancy period (29%) and at birth (27%) than mothers of the control group (13% and 7%, respectively, *p* = .015). Additionally, more cases required professional assistance from the doctors than the controls (17% vs. 4%, *p* = .008; Table 1).

**Table 1:** Characteristics of participants

	NNJ cases	Control group	*p* value*
	*n* = 57	*n* = 112	
Clinical history, *n* (%)			
Phototherapy + saline	26 (46)	—	—
Phototherapy + albumin	31 (54)	—	—
Obstetric history, *n* (%)			
Maternal employment			
Employed	31 (62.0)	43 (43.9)	**.037**
Not employed	19 (38.0)	55 (56.1)	
Pregnancy status			
Abnormal	14 (29.2)	13 (12.8)	**.015**
Normal	34 (70.8)	89 (87.3)	
Place of delivery			
Hospital	27 (54.0)	66 (66.7)	.132
Home	23 (46.0)	33 (33.3)	
Nature of delivery			
Abnormal	13 (26.5)	7 (7.1)	**.001**
Normal	36 (73.5)	92 (92.9)	
Who assisted in delivery			
Doctor	10 (17.5)	4 (3.57)	**.008**
Nurse	15 (26.3)	28 (25.0)	
Traditional birth attendant	6 (10.5)	8 (7.1)	
Relative/other attendants	26 (45.6)	72 (64.3)	
Perinatal history, *n* (%)			
Breathing problems	4 (8.0)	3 (3.0)	.167
Crying problems	19 (18.8)	16 (32.0)	.071
Breastfeeding problems	24 (48.0)	30 (29.7)	**.027**
Presence of fits	3 (6.3)	0 (0.0)	**.017**
Previous hospital admission	50 (100.0)	11 (11.6)	**<.001**
Problem after delivery	24 (48.0)	5 (5.3)	**<.001**
Mother education level, *n* (%)			
No education	17 (29.8)	51 (45.5)	.140
Primary school	36 (63.2)	54 (48.1)	
Secondary school	4 (7.0)	7 (6.3)	

Abbreviation: NNJ, neonatal jaundice.

Bold values indicate significant differences between the groups

* Pearson's χ^2^.

### 3.4 Perinatal history

Compared with controls, significantly more of the cases had breastfeeding problems, previous hospital admissions, or health problems after birth. Table 1 presents a summary of these findings. Generally, the cases had poorer health outcomes than the controls.

### 3.5 Developmental outcome

Children who survived NNJ scored lower than controls on both developmental outcome measures. Specifically, the cases scored significantly lower than controls on language functioning ([*M* = 6.5, *SD* = 4.3 vs. *M* = 8.9, *SD* = 4.6], *p* < .001); psychomotor functioning ([*Mdn* = 31, IQR = 17, 34 vs. *Mdn* = 31.0, IQR = 22.0, 44.0], Mann–Whitney *U* = 4,122, *p* = .002); and socio‐emotional functioning ([*Mdn* = 34.0, IQR = 30.0, 37.0 vs. *Mdn* = 30.0, IQR = 27.0–33.0], Mann–Whitney *U* = 4,289, *p* < .001; Table 2). There were no significant correlations between sepsis and psychomotor (*r*_pb_ = −.2, *p* = .214), language (*r*_pb_ = −.1, *p* = .510), and socio‐emotional functioning (*r*_pb_ = .0, *p* = .916). Additionally, there were no differences in the developmental outcomes (*p* > .05) between children who had both NNJ and sepsis versus those who had NNJ only (Table 2).

**Table 2:** Developmental outcomes in sepsis + NNJ versus NNJ only

	Sepsis + NNJ group	NNJ group without sepsis	Control group	Sepsis + NNJ versus NNJ only	Comparison among three groups	Total NNJ versus control group
	*N* = 13	*N* = 44	*N* = 112	*p* value	*p* value	*p* value
Developmental features, *M* (*SD*)						
Psychomotor, *Mdn* (IQR)^a^	20.0 (7.0–22.0)	25.1 (19–36)	31 (22–44)	0.066	**0.001**	**0.002** c
Language, *M* (*SD*)^b^	6.3 (4.2)	6.6 (4.3)	8.9 (4.6)	0.977	0.247	**<0.001** d
Socio‐emotional status, *Mdn* (IQR)^a^	30.0 (27.0–32.0)	29.0 (26.0–34.0)	34 (30–37)	1.000	**0.001**	**<0.001** c

*Note.* Bolded values indicate significant differences among/between groups.

Abbreviations: IQR, interquartile range; NNJ, neonatal jaundice.

a Kruskal–Wallis test.

b Analysis of variance.

c *t* test.

d Mann–Whitney *U* test.

### 3.6 Risk factors associated with poor developmental outcomes in NNJ

Six participants had a neurological impairment (motor impairment, seizure disorders, cerebral palsy, and abnormality in optokinetic nystagmus). The results of the multiple regression indicate that significant (*p* < .020) and medium to large portions (34–64%) of the variance in all the three developmental outcomes of NNJ were jointly associated with the identified risk domains. Home births were associated with a significant reduction of 4.5 (*p* = .012) and 10.2 (*p* = .040) points in scores in language and socio‐emotional functioning, respectively. Lack of maternal occupation and education was associated with a significant reduction of 8.5 (*p* = .040) and 15.2 (*p* = .000) points in scores in psychomotor functioning. Having an unskilled birth attendant was associated with a significant reduction of 4.6 (*p* = .000) points in scores in language functioning, whereas having feeding difficulties after birth was associated with a reduction of 8.2 (*p* = .030) points in scores in psychomotor functioning in the NNJ cases (Table 3).

**Table 3:** Risk factors associated with poor developmental outcomes in NNJ as identified with multiple regression analysis

	Psychomotor		Language		Socio‐emotional functioning	
Risk factors	Coefficient (95% CI)	*p* value	Coefficient (95% CI)	*p* value	Coefficient (95% CI)	*p* value
Hospital delivery (reference)	—	—	—	—	—	—
Giving birth at home	−11.5 [−24.9, 1.9]	.090	−4.5 [−8.1, −1.1]	**.012**	−10.2 [−19.5, −0.8]	**.040**
Normal delivery (reference)	—	—	—	—	—	—
Abnormal delivery	−3.3 [−14.6, 8.1]	.560	−1.8 [−5.0, 1.4]	.260	−0.1 [−7.7, −7.5]	.980
Maternal occupation	−8.5 [−16.6, −0.3]	**.040**	−1.5 [−4.7, 1.6]	.330	−0.6 [−6.2, 5.1]	.830
Who assisted in delivery						
Unskilled birth attendant (reference)	—	—	—	—	—	—
Traditional birth attendant	−4.1 [−13.6, 5.5]	.390	1.7 [−2.5, 5.8]	0.820	5.5 [−1.6, 12.5]	.120
Nurse	−19.7 [−31.5, −7.9]	.120	−4.6 [−7.9, −1.3]	**.000**	−5.2 [−14.3, 3.8]	.250
Doctor	−4.8 [−20.4, 10.9]	.320	−1.4 [−6.9, 4.1]	.610	−5.6 [−17.2, 6.0]	.330
Mother level of education						
No education (reference)	—	—	—	—	—	—
Primary	−15.2 [−25.1, −5.3]	**.000**	−2.2 [−5.1, 0.7]	.330	−3.9 [−11.8, 4.1]	.540
Secondary	−4.3 [−17.8, 9.2]	.520	−2.1 [−8.3, 4.1]	.240	−7.7 [−20.8, 5.3]	**.090**
Perinatal problems						
Problems after delivery	−2.5 [−13.1, 8.2]	.640	2.0 [−1.6, 5.6]	.260	0.0 [−7.2, 7.3]	**.010**
Crying problems	2.2 [−5.4, 9.9]	.560	−3.3 [−5.7, −0.9]	**.010**	−4.6 [−9.8, 0.6]	.080
Feeding problems	−8.2 [−15.5, −0.9]	**.030**	−1.3 [−4.0, 1.4]	.330	−0.9 [−7.8, 6.1]	.810
Breathing problems	−6.9 [−21.4, 7.6]	.340	−2.5 [−7.2, 2.2]	.280	6.2 [−3.8, 16.2]	.220
Female (reference)	—	—	—	—	—	—
Male	−2.5 [−10.2, 5.3]	.520	−2.5 [−5.1, 0.1]	**.060**	−1.4 [−6.1, 3.3]	.550
Height‐for‐age	−9.6 [−17.7, − 1.4]	**.023**	−1.3 [−4.7, 2.1]	.440	−3.9 [−7.6, −0.2]	**.040**

Abbreviations: CI, confidence interval; NNJ, neonatal jaundice.

Bold values indicate significant differences between the groups

## 4 DISCUSSION

The purpose of this study was to establish developmental outcomes among infants who had survived NNJ and, in particular, determine whether neonatal sepsis aggravated the poor developmental outcomes. We observed that children who survived NNJ experienced global developmental delay. NNJ associated with sepsis did not appear to have a worse outcome. Additionally, home birth, unskilled birth attendant, low maternal level of education, and having feeding problems were the main risk factors associated with poor developmental outcomes in survivors of NNJ.

### 4.1 Developmental outcomes in NNJ

Our findings suggest that children with NNJ have poor developmental outcomes compared with their healthy counterparts, but that sepsis is not associated with additional impairments. This finding corroborates earlier findings that indicate that different domains of development are affected in children who survived NNJ (Chen et al., 2014; Gordon et al., 2005; Naeye, 1978). Chen et al. (2014) reported that neonates who developed NNJ were at a higher risk of developing speech or language problems (Chen et al., 2014), whereas Gordon et al. (2005) assert that infants who survived NNJ were likely to have neurological impairment (Gordon et al., 2005). To the best of our knowledge, there are no studies that have investigated the association between NNJ and poor socio‐emotional functioning in children.

Our study differs from Amin, Prinzing, and Myers (2009), who did not find any significant relationship between NNJ and language. The difference in findings between these two studies could be due to differences in methodology and participants' characteristics. In the Amin et al. study, only premature infants with hyperbilirubinaemia were included as opposed to the current study where neonates of any gestational age and a TSB level of >250 µmol/L qualified for inclusion.

Poor developmental outcomes are likely to be a manifestation of hyperbilirubinaemia neurotoxicity, which causes brain injury to specific brain regions such as the frontal lobe, the cerebellum, basal ganglia, and the prefrontal cortex areas that are associated with language, motor, and socio‐emotional functioning, respectively (Mwaniki, Atieno, Lawn, & Newton, 2012; Wusthoff & Loe, 2015).

### 4.2 Risk factors to poor outcomes in children who survived NNJ

Several risk factors were associated with poor developmental outcomes in children who survived NNJ. Our findings suggest that home birth, help from an unskilled birth attendant, low maternal level of education, and having feeding problems were associated with poor language, psychomotor, and socio‐emotional functioning. Low maternal education has been associated with poor child development in some studies (Khan, Soomro, Soomro, & Hafeez, 1994; Mbagaya & Odhiambo, 2005; Webair & Bin‐Gouth, 2013). Researchers postulate that mothers with low education levels may not be able to adequately stimulate their children at home, thus impairing child development (Amin et al., 2009). Besides, educated mothers are more likely to seek help whenever they feel their children need medical care than mothers with no education. These health‐seeking behaviours may potentially reduce the negative impact of NNJ on their children's developmental outcome (Khan et al., 1994).

Our study suggests that neonates who had feeding problems have poor developmental outcomes. This finding corroborates those by Wolke, Schmid, Schreier, and Meyer (2009), who reported that feeding problems in neonates might result in poor developmental outcomes. Poor feeding may result in impaired growth and development of critical brain areas such as the frontal lobe and the cerebellum that are important in speech and motor development (Wolke et al., 2009). However, poor feeding may be a manifestation of developmental impairment.

Lastly, our study found out that home births assisted by unskilled persons were associated with poor developmental outcomes. According to Moindi, Ngari, Nyambati, and Mbakaya (2015), mothers in low resource settings deliver at home due to the inaccessibility of health facilities. Our findings support those of Kariuki et al. (2017), who report that home births may result in perinatal complications, which lead to emotional and behavioural problems in children. Similarly, Mung'ala‐Odera et al. (2006) reported that mothers who were assisted by unskilled personnel had higher chances of having children with poor developmental outcomes. Home deliveries assisted by an unskilled person may lead to birth complications such as birth trauma that may further affect the child's development. Home deliveries may also be related to the low socio‐economic status, which contributes to the poor developmental and nutritional outcome.

### 4.3 Study limitations and strengths

Limitations of this study include the fact that the study was only a 12‐month follow‐up; thus, we are not able to infer to the long‐term developmental outcomes in children who survived NNJ. Although our study identified risk factors associated with poor developmental outcomes in NNJ survivors, it is beyond the scope of this study to infer causal relationships between NNJ, the associated risk factors, and poor developmental outcomes. The neonates may have developed other infections at admission, which may not have been possible to investigate using blood culture and may require further laboratory investigations using advanced technology. It is possible that at 1‐year, children may not have developed complications such as cerebral palsy, and the tools used in this study could not provide a diagnosis for cerebral palsy.

Therefore, future longitudinal studies using predictive assessment tools for cerebral palsy should incorporate diagnostic tools such as the Hammersmith Infant Neurological Examination. Moreover, this study did not examine other potential household‐level predictors of childhood outcomes, including the quality and quantity of stimulation at home and maternal/caregiver mental health, and perinatal factors such as gestational age, among others. Future studies could potentially investigate the role of these variables in predicting developmental outcomes in infancy.

Additionally, it was not possible to control for other child developmental predictors such as NNJ severity (all NNJ cases had severe hyperbilirubinaemia). The main strength of this study is the relatively large sample size to detect clinically significant differences in developmental outcomes between the cases and controls. Moreover, the use of locally validated measures of developmental outcomes provides greater confidence in interpreting the results of this study.

## 5 CONCLUSIONS AND RECOMMENDATIONS

The findings of this study have important implications for intervention and research focusing on developmental outcomes in children who survived neonatal insults. The study suggests that children who survived NNJ are likely to have poor developmental outcomes, which manifest early in life, and this is associated with poor socio‐economic conditions. These developmental needs may be amenable to intervention. Therefore, there is a need for adequate treatment and care for neonates who develop NNJ to prevent the adverse effect of NNJ on their reaching of developmental milestones. Given the strong evidence based on the potential positive impact of early psychosocial stimulation on developmental outcomes of at‐risk children, our results suggest the need for the implementation of early intervention measures to enhance outcomes among survivors of NNJ. The development of children with NNJ needs to be monitored after discharge from the hospitals and at subsequent years. Moreover, future empirical studies should focus on understanding the long‐term outcomes beyond infancy period. An understanding of the long‐term outcomes beyond infancy period will give insights into causality and potential interventions required.

## CONFLICT OF INTEREST

No competing interests were disclosed.

## AUTHOR CONTRIBUTIONS

M. M. and C. R. N. conceptualized the study. M. M., R. K., and C. R. N. designed the study and prepared the tools for the study. P. M. assisted in retrieving the participants from KHDSS and in data management. D. N. M. entered the data, conducted the data analysis, and drafted the manuscript. C. R. N., M. M., R. K., A. A., H. M. K., A. L. G., and P. M. reviewed the manuscript, critically interpreted the results and contributed to the writing of the manuscript.

## ETHICAL APPROVAL

Ethical approval for this study was granted by the Kenya Medical Institute Scientific and Ethics Review Unit (SERU); SERU protocol number 1592.

## References

[cch12750-bib-0001] Abubakar, A. , Holding, P. , van Baar, A. , Newton, C. R. J. C. , & van de Vijver, F. J. R. (2008). Monitoring psychomotor development in a resource limited setting: an evaluation of the Kilifi Developmental Inventory. Annals of Tropical Paediatrics, 28, 217–226. 10.1179/146532808X335679 18727851 PMC3908377

[cch12750-bib-0002] Abubakar, A. , Holding, P. , Van De Vijver, F. , Bomu, G. , & Van Baar, A. (2010). Developmental monitoring using caregiver reports in a resource‐limited setting: The case of Kilifi, Kenya. Acta Paediatrica, International Journal of Paediatrics, 99, 291–297. 10.1111/j.1651-2227.2009.01561 PMC2814084 20353499

[cch12750-bib-0003] Abubakar, A. , Van de Vijver, F. , Van Baar, A. , Mbonani, L. , Kalu, R. , Newton, C. , & Holding, P. (2008). Socioeconomic status, anthropometric status, and psychomotor development of Kenyan children from resource‐limited settings: A path‐analytic study. Early Human Development, 84, 613–621. 10.1016/j.earlhumdev.2008.02.003 18499363 PMC4825882

[cch12750-bib-0004] Amin, S. B. , Prinzing, D. , & Myers, G. (2009). Hyperbilirubinemia and language delay in premature infants. Changes, 123, 327–331. 10.1016/j.biotechadv.2011.08.021 PMC3264948 19117899

[cch12750-bib-0005] Chen, M.‐H. , Su, T.‐P. , Chen, Y.‐S. , Hsu, J.‐W. , Huang, K.‐L. , Chang, W.‐H. , & Bai, Y.‐M. (2014). Is neonatal jaundice associated with autism spectrum disorder, attention deficit hyperactivity disorder, and other psychological development? A nationwide prospective study. Research in Autism Spectrum Disorders, 8, 625–632. 10.1016/j.rasd.2014.03.006

[cch12750-bib-0006] English, M. , Ngama, M. , Musumba, C. , Wamola, B. , Bwika, J. , Mohammed, S. , & McHugh, K. (2003). Causes and outcome of young infant admissions to a Kenyan district hospital. Archives of Disease in Childhood, 88(5), 438–443. 10.1136/adc.88.5.438 12716721 PMC1719579

[cch12750-bib-0007] Garcia, F. J. , & Nager, A. L. (2002). Jaundice as an early diagnostic sign of urinary tract infection in infancy. Pediatrics, 109(5), 846–851. 10.1542/peds.109.5.846 11986445

[cch12750-bib-0008] Gordon, A. L. , English, M. , Dzombo, J. T. , Karisa, M. , & Newton, C. R. J. C. (2005). Neurological and developmental outcome of neonatal jaundice and sepsis in rural Kenya. Tropical Medicine and International Health, 10, 1114–1120. 10.1111/j.1365-3156.2005.01496.x 16262736

[cch12750-bib-0009] Kariuki, S. M. , Abubakar, A. , Kombe, M. , Kazungu, M. , Odhiambo, R. , Stein, A. , & Newton, C. R. J. C. (2017). Burden, risk factors, and comorbidities of behavioural and emotional problems in Kenyan children: A population‐based study. The Lancet Psychiatry, 4, 136–145. 10.1016/S2215-0366(16)30403-5 28137381 PMC5285446

[cch12750-bib-0010] Khan, Z. , Soomro, G. Y. , Soomro, S. , & Hafeez, S. (1994). Mother's education and utilisation of health care services in Pakistan [with comments]. The Pakistan Development Review *,* 33(4), 1155–1166. 12346196

[cch12750-bib-0011] Linder, N. , Yatsiv, I. , Tsur, M. , Matoth, I. , Mandelberg, A. , Hoffman, B. , & Tamir, I. (1988). Unexplained neonatal jaundice as an early diagnostic sign of septicemia in the newborn. Journal of Perinatology: Official Journal of the California Perinatal Association, 8(4), 325–327. 3236101

[cch12750-bib-0012] Magai, D. N. , Mwaniki, M. , Abubakar, A. , Kalu, R. , Mwangi, P. , Koot, H. M. , & Newton, C. R. (2019). A randomized control trial of phototherapy and albumin versus phototherapy and saline in Kilifi, Kenya. BMC Research Notes, 12(1), 617 10.1186/s13104-019-4632-2 31547861 PMC6757356

[cch12750-bib-0013] Maimburg, R. D. , Bech, B. H. , Væth, M. , Møller‐Madsen, B. , & Olsen, J. (2010). Neonatal jaundice, autism, and other disorders of psychological development. Pediatrics, 126(5), 872–878. 10.1542/peds.2010-0052 20937652

[cch12750-bib-0014] Mbagaya, G. M. , & Odhiambo, M. O. (2005). Mother's health seeking behaviour during child illness in a rural western Kenya community. African Health Sciences, 5(4), 322–327. 10.5555/afhs.2005.5.4.322 16615844 PMC1831955

[cch12750-bib-0015] Moindi, R. O. , Ngari, M. M. , Nyambati, V. C. S. , & Mbakaya, C. (2015). Why mothers still deliver at home: Understanding factors associated with home deliveries and cultural practices in rural coastal Kenya, a cross‐section study. BMC Public Health, 16(114), 1–8. 10.1186/s12889-016-2780-z PMC4738797 26842657

[cch12750-bib-0016] Mung'ala‐Odera, V. , Meehan, R. , Njuguna, P. , Mturi, N. , Alcock, K. J. , & Newton, C. R. J. C. (2006). Prevalence and risk factors of neurological disability and impairment in children living in rural Kenya. International Journal of Epidemiology, 35(3), 683–688. 10.1093/ije/dyl023 16492712

[cch12750-bib-0017] Mwaniki, M. K. , Atieno, M. , Lawn, J. E. , & Newton, C. R. J. C. (2012). Long‐term neurodevelopmental outcomes after intrauterine and neonatal insults: A systematic review. The Lancet, 379, 445–452. 10.1016/S0140-6736(11)61577-8 PMC3273721 22244654

[cch12750-bib-0018] Naeye, R. L. (1978). Amniotic fluid infections, neonatal hyperbilirubinemia, and psychomotor impairment. Pediatrics, 62(4), 497–503. 714581

[cch12750-bib-0019] Ogunlesi, T. , Dedeke, I. , Adekanmbi, A. , Fetuga, M. , & Ogunfowora, O. (2007). The incidence and outcome of bilirubin encephalopathy in Nigeria: A bi‐centre study. Nigerian Journal of Medicine: Journal of the National Association of Resident Doctors of Nigeria, 16(4), 354–359. 18080595

[cch12750-bib-0020] Olusanya, B. , Akande, A. , Emokpae, A. , & Olowe, S. (2009). Infants with severe neonatal jaundice in Lagos, Nigeria: Incidence, correlates and hearing screening outcomes. Tropical Medicine & International Health, 14(3), 301–310. 10.1111/j.1365-3156.2009.02223.x 19187520

[cch12750-bib-0021] Owa, J. , & Dawodu, A. (1990). Neonatal jaundice among Nigerian preterm infants. West African Journal of Medicine, 9(4), 252–257. 2083201

[cch12750-bib-0022] Peters, D. H. , Garg, A. , Bloom, G. , Walker, D. G. , Brieger, W. R. , & Rahman, M. H. (2008). Poverty and access to health care in developing countries. Annals of the new York Academy of Sciences, 1136(1), 161–171. 17954679 10.1196/annals.1425.011

[cch12750-bib-0023] Poole, K. , Wright, L. L. , Stoll, B. J. , Ehrenkranz, R. A. , Carlo, W. A. , Shankaran, S. , … Perritt, R. (2003). Association between peak serum bilirubin and neurodevelopmental outcomes in extremely low birth weight infants. Pediatrics, 112(4), 773–779. 10.1542/peds.112.4.773 14523165

[cch12750-bib-0024] Rennie, J. , Burman‐Roy, S. , & Murphy, M. S. (2010). Neonatal jaundice: Summary of NICE guidance. BMJ, 340, 1190–1192. 10.1136/bmj.c2409 20484363

[cch12750-bib-0025] Shahian, M. , Rashtian, P. , & Kalani, M. (2012). Unexplained neonatal jaundice as an early diagnostic sign of urinary tract infection. International Journal of Infectious Diseases, 16(7), 487–490. 10.1016/j.ijid.2012.02.011 22512850

[cch12750-bib-0026] Slusher, T. , & Olusanya, B. (2012). Neonatal jaundice in low‐and middle‐income countries In Care of the jaundiced neonate (pp. 263–273). New York: McGraw‐Hill.

[cch12750-bib-0027] StataCorp . (2017) Stata statistical software: Release 15., College Station, TX: StataCorp LLC.

[cch12750-bib-0028] Stoll, B. J. , Hansen, N. I. , Adams‐Chapman, I. , Fanaroff, A. A. , Hintz, S. R. , Vohr, B. , & Network, H. D. N. R. (2004). Neurodevelopmental and growth impairment among extremely low‐birth‐weight infants with neonatal infection. JAMA, 292(19), 2357–2365. 10.1001/jama.292.19.2357 15547163

[cch12750-bib-0029] World Health Organization (2007). WHO Anthro for personal computers manual. Software for Assessing Growth and Development of the World's Children. Geneva: WHO.

[cch12750-bib-0030] World Health Organization (2013). Pocketbook of hospital care for children: Guidelines for the management of common childhood illnesses. World Health Organization. 1–412. 24006557

[cch12750-bib-0031] World Health Organization . (1999). Integrated Management of Childhood Illness (IMCI): Management of childhood illness in developing countries: Rationale for an integrated strategy. Retrieved from http://www.who.int/child-adolescent-health/New_Publications/IMCI/WHO_CHS_CAH_98.1/Rev99.Apdf.

[cch12750-bib-0032] Webair, H. H. , & Bin‐Gouth, A. S. (2013). Factors affecting health seeking behavior for common childhood illnesses in Yemen. Patient Preference and Adherence, 7, 1129–1138. 24187490 10.2147/PPA.S51124 PMC3810343

[cch12750-bib-0033] Wolf, M. , Beunen, G. , Casaer, P. , & Wolf, B. (1997). Extreme hyperbilirubinaemia in Zimbabwean neonates: Neurodevelopmental outcome at 4 months. European Journal of Pediatrics, 156(10), 803–807. 10.1007/s004310050718 9365074

[cch12750-bib-0034] Wolf, M.‐J. , Wolf, B. , Beunen, G. , & Casaer, P. (1999). Neurodevelopmental outcome at 1 year in Zimbabwean neonates with extreme hyperbilirubinaemia. European Journal of Pediatrics, 158(2), 111–114. 10.1007/s004310051029 10048606

[cch12750-bib-0035] Wolke, D. , Schmid, G. , Schreier, A. , & Meyer, R. (2009). Crying and feeding problems in infancy and cognitive outcome in preschool children born at risk: A prospective population study. Journal of Developmental & Behavioral Pediatrics, 30(3), 226–238. 10.1097/DBP.0b013e3181a85973 19433987

[cch12750-bib-0036] Wusthoff, C. J. , & Loe, I. M. (2015). Impact of bilirubin‐induced neurologic dysfunction on neurodevelopmental outcomes. Seminars in Fetal & Neonatal Medicine, 20(1), 52–57. 10.1016/j.siny.2014.12.003 25585889 PMC4651619

